# Using Virtual Reality to Reduce Stress in Adolescents: Mixed Methods Usability Study

**DOI:** 10.2196/49171

**Published:** 2024-04-22

**Authors:** Elin A Björling, Jennifer Sonney, Himanshu Zade, Sofia Rodriguez, Michael D Pullmann, Soo Hyun Moon

**Affiliations:** 1 Human Centered Design and Engineering University of Washington Seattle, WA United States; 2 Child, Family, and Population Health Nursing University of Washington Seattle, WA United States; 3 Psychiatry & Behavioral Sciences University of Washington Seattle, WA United States

**Keywords:** virtual reality, adolescents, perceived stress, participatory design, depression

## Abstract

**Background:**

Adolescent mental health is a national mental health emergency amid surging rates of anxiety and depression. Given the scarcity and lack of scalable mental health services, the use of self-administered, evidence-based technologies to support adolescent mental health is both timely and imperative.

**Objective:**

The goal of this study was 2-fold: (1) to determine the feasibility, usability, and engagement of a participatory designed, nature-based virtual reality (VR) environment and (2) to determine the preliminary outcomes of our self-administered VR environment on depression, mindfulness, perceived stress, and momentary stress and mood.

**Methods:**

We conducted a within-person, 3-week, in-home study with a community-based sample of 44 adolescents. Participants completed surveys of perceived stress, depression, cognitive fusion, and mindfulness at intake, postintervention, and a 3-week follow-up. Participants were invited to use a nature-based, VR environment that included 6 evidence-based activities 3 to 5 times per week. They completed momentary stress and mood surveys 5 times each day and before and after each VR session. Postintervention, participants completed surveys on system and intervention usability and their experiences with using the VR system. Quantitative data were analyzed using descriptive statistics and mixed effects modeling to explore the effect of the VR environment on stress. Qualitative data were analyzed using collaborative thematic analysis.

**Results:**

Participants’ use of the VR environment ranged from 1 session to 24 sessions (mean 6.27 sessions) at home over a 3-week period. The 44 participants completed all study protocols, indicating our protocol was feasible and the VR environment was engaging for most. Both the use of the VR system and novel VR intervention received strong usability ratings (mean 74.87 on the System Usability Scale). Most teens indicated that they found the tool to be easily administered, relaxing, and helpful with stress. For some, it offered space to process difficult emotions. The themes *calm*, *regulating*, and *forget about everything* resulted from open-ended exit interview data. Although the Relaxation Environment for Stress in Teens (RESeT) did not significantly affect repeated survey measurements of depression, mindfulness, nor cognitive fusion, it did positively affect momentary mood (pre-intervention: 10.8, post-intervention: 12.0, *P*=.001) and decrease momentary stress (pre-intervention: 37.9, post-intervention: 20.6, *P*=.001*)*. We found a significant reduction in within-day momentary stress that strengthened with increased VR use over time during the study period (*P*=.03).

**Conclusions:**

These preliminary data inform our own VR environment design but also provide evidence of the potential for self-administered VR as a promising tool to support adolescent mental health. Self-administered VR for mental health may be an effective intervention for reducing adolescent stress. However, understanding barriers (including disengagement) to using VR, as well as further encouraging participatory design with teens, may be imperative to the success of future mental health interventions.

## Introduction

It is good to like, to have the brain stop for a second and focus on small things.

P98, girl, 16 years old, exit interview

### The Potential for Virtual Reality in Adolescent Mental Health

In a 2018 American Psychological Association survey [1], teens reported worse mental health and higher levels of anxiety and depression than all other age groups. Increased stress causes adverse mental and physical outcomes, including anxiety and depression [2]. However, despite effective, evidence-based treatments for adverse outcomes associated with stress [3], only one-half of teens will receive mental health services due to mental health provider shortages and other barriers to accessing care [4,5]. Even worse, evidence-based therapies are often inaccessible due to cost, time, or the need for a trained interventionist [6]. Therefore, a self-administered, technology-based solution could increase accessibility and scalability of these therapies.

Virtual reality (VR)–based serious games (SGs) for mental health present an opportunity for the translation of effective mental health strategies to an engaging platform [7,8]. VR consists of a head-mounted display that displays simulated environments for exploration and interaction. The immersive and intuitive experience of VR makes it an optimal platform for delivering self-administered SG health interventions for adolescents [9]. Additionally, VR-based SGs are scalable, which could increase accessibility to evidence-based mental health care. VR has been successfully deployed in treating an array of health conditions in adults, including posttraumatic stress disorder [10], phobias [11], and perceived stress in military personnel [12].

The use of VR as an intervention platform for adolescent mental health is an emerging area of inquiry [13,14]. VR has been shown to be acceptable and effective in treating procedural pain, headaches, and public speaking anxiety in adolescents [15-18]. In addition, Björling et al [19] found that a nature-based environment reduced stress in teens and that teens will self-administer VR therapeutically. Building on these findings, it has been suggested that existing evidence-based mental health therapies, such as cognitive behavioral theory (CBT), could be translated into a VR environment as an innovative approach to delivering scalable mental health interventions [20]. Although a recent systematic review of clinical trials of mostly computer-based video games found CBT SGs to be more effective than no intervention, true efficacy was clouded by study rigor, and none of the CBT games were VR [21]. In a systematic review of VR environments intended to reduce pain and anxiety in children and adolescents, Ahmadpour et al [22] proposed that future VR interventions explore skill building and provide dynamic feedback to participants to enable them to be an active participant in managing their own care.

### The Importance of Participatory Design

Participatory design (PD) is an approach in which the people who are “destined to use the system play a critical role in designing it” [23]. In PD, the goal is not to simply build systems that address the needs and wants of people. Rather, the hallmark of PD is to establish cooperative and collaborative design relationships that engage users throughout the iterative design process. Research results are often collaboratively interpreted by designer-researchers and the participants who will use the design. For this very reason, gathering data continually during the design and testing phases of development is essential. Such a collaborative approach is thought to result in solutions that address real-world needs and priorities in people’s lives [24,25].

Engaging teens in PD is rare in the design and development of VR, though it is a successful methodology for working with teens in relation to mental health [26-28]. PD is an appropriate approach for designing new technologies with teens due to its meaningful engagement of participants throughout the design process [29,30]. However, engagement is highly variable, and the methods are often iterative and flexible [26]. Examples of engaging teens in co-design for mental health include suicide prevention through social media [27] and stress reduction via a social robot [28]. In fact, co-design with youth specifically for VR has also shown to be successful. Realpe et al [31] engaged youth in the co-design of a virtual environment as a social cognition intervention for people with a first episode of psychosis. Björling et al [32] successfully engaged teens in the design of a VR environment aimed to reduce stress.

### Our Motivation: A Study of Usability and Experience

As a technology, VR holds the potential to provide immersive experiences and skills training to reinforce evidence-based mental health practices. However, in order to be effective, it must be engaging and usable by teens. Therefore, we designed and developed our VR environment, Relaxation Environment for Stress in Teens (RESeT), using a human-centered, PD approach. We engaged adolescents in each stage of development to ensure usefulness and maximize engagement. Equally important was to empirically measure usability and user experience. Therefore, in our pilot study of our novel VR environment, we explored 2 aims and associated research questions (RQs).

### Aim 1

The first aim was to measure the implementation outcomes (feasibility, acceptability, appropriateness, usability, and engagement) of a participatory designed, nature-based VR environment.

- RQ1: How did teens use RESeT, and how did they rate its implementation?
- RQ2: What is the experience of using RESeT?

### Aim 2

The second aim was to determine the preliminary mental health outcomes of our self-administered VR environment on depression, mindfulness, perceived stress, and momentary stress and mood.

- RQ3: What effect does RESeT use have on retrospective stress (Perceived Stress Scale [PSS]), depression (Patient Health Questionnaire 9 [PHQ-9]), cognitive fusion (Cognitive Fusion Questionnaire [CFQ]), or mindfulness (Mindfulness Attention and Awareness Scale [MAAS])?
- RQ4: What effect does RESeT use have on momentary stress and mood?
  RQ4a: Do baseline depression, mindfulness, and stress moderate the effect of [VR environment] on momentary stress over the 3-week intervention?
  RQ4b: Does dosage (frequency and duration of VR use) moderate momentary stress over the 3-week intervention?

## Methods

### Study Timeline

The prospective, within-person study design utilized multiple layers of measurement. Participants completed surveys at intake, exit, and follow-up as well as within-day momentary measurements of stress and mood and pre- and post-VR measurements. See Table 1 for a summary of the participant activities and measurement over time.

**Table 1:** Study measurement timeline.

Study activity			Research question (RQ)		Intake		5 times per day during the 3-week intervention		Pre/post-VR^a^ use		Exit (at 3 weeks)		Follow-up (at 7 weeks)
**Survey instruments**													
	PHQ-9^b^ (depression)	RQ3		✓						✓		✓	
	PSS^c^ (retrospective stress)	RQ3		✓						✓		✓	
	CFQ^d^ (cognitive fusion)	RQ3		✓						✓		✓	
	MAAS^e^ (mindfulness/attention)	RQ3		✓						✓		✓	
	SUS^f^/IUS^g^ (usability)	RQ1								✓			
	IAM^h^, FIM^i^, AIM^j^ (appropriateness, feasibility, acceptability of intervention)	RQ1								✓			
	Interview (user experience)	RQ2								✓			
**Momentary instruments**													
	Stress	RQ4				✓		✓					
	Sadness	RQ4				✓							
	Affect	RQ4						✓					
	Comfort	RQ4						✓					

^a^VR: virtual reality.

^b^PHQ-9: Patient Health Questionnaire 9.

^c^PSS: Perceived Stress Scale.

^d^CFQ: Cognitive Fusion Questionnaire.

^e^MAAS: Mindfulness Attention and Awareness Scale.

^f^SUS: System Usability Scale.

^g^IUS: Intervention Usability Questionnaire.

^h^IAM: Acceptability of Intervention Measure.

^i^FIM: Feasibility of Intervention Measure.

^j^AIM: Appropriateness of Intervention Measure.

### The Development of a Relaxation Environment for Teen Stress

The novel VR RESeT was developed in partnership with teens and the Seattle Public Library. Design of RESeT began by eliciting teen ideas and design principles through PD sessions spanning 2 years with approximately 60 teens at local library sites. Some of our design-session studies are described in a previous publication [32]. Based upon teen preferences, RESeT was designed as an open, explorable world filled with nature, animals, and calming activities. The activities in RESeT incorporate evidence-based mental health activities stemming from dialectical behavioral therapy [33], acceptance and commitment therapy [34], and mindfulness-based stress reduction for teens [35]. Each of these therapies have been shown to be very effective in adolescents. Although such self-administered exercises are typically administered in workbooks and worksheets, we incorporated them into the immersive and interactive VR world with teens as our co-designers.

As an example, teens experience “defusion” from negative emotions in the VR environment by placing negative words into a paper boat and releasing the boat out onto a river. As the boat floats away from them, they can reflect on their ability to release negative emotions rather than hold them tightly or be “fused” with them. This process is called *cognitive defusion* [36]. Another example in the environment is building a teen’s capacity for mindfulness through an interaction that invites teens to listen carefully for different birds and look to find where their song is coming from. Integrating these activities was iteratively refined through a series of design sessions (37 teens) and usability testing sessions (9 teens) resulting in a fully functional RESeT comprised of 6 evidence-based interactions. In addition, the game was designed with arm-swing locomotion where the player swings their arms back and forth to create a natural walking pace in VR. The faster they swing their arms, the faster they move through the environment. Not only is this type of locomotion naturalistic, it has also been shown to improve mood [12].

The RESeT is a natural environment set in winter, consisting of a mountainous border surrounding a snow-covered, tree-filled meadow. A river runs through the meadow, providing a natural border to create an open world feeling for player exploration. A riverboat provides players with a relaxing space in which they can explore and participate in various activities without having a time limit or required objective. Nature sounds and ambient music play in the background through the experience.

**Figure 1:**
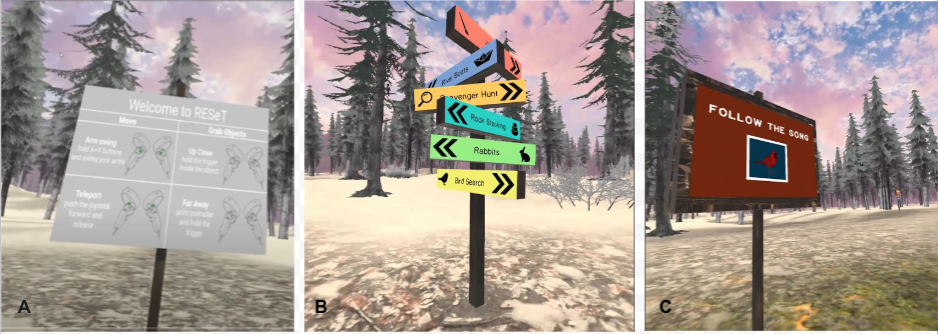
Examples of the (A) controller, (B) wayfinding, and (C) activity navigation.

Players start the game at a home base location in the middle of the map. See Figure 1 for an illustration.

**Figure 2:**
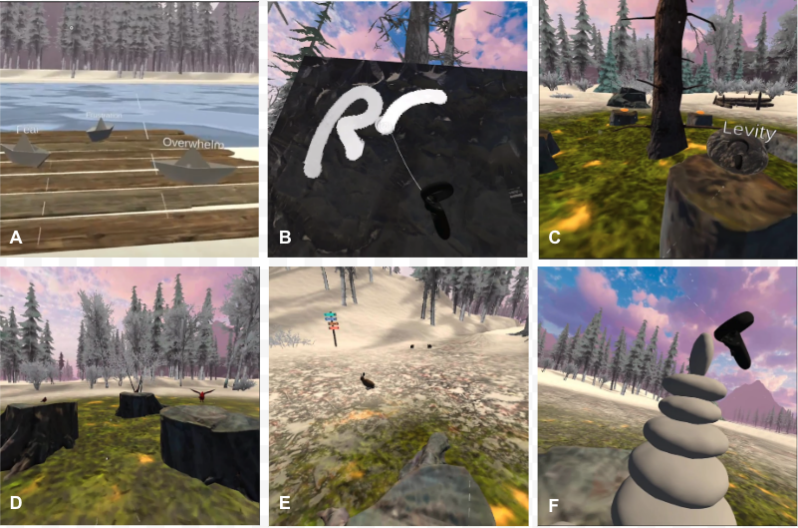
Screenshots of gameplay illustrating each of the 6 activities: (A) riverboat, (B) painting, (C) scavenger hunt, (D) bird search, (E) rabbit hole, (F) rock stacking.

The environment is designed for seated or standing gameplay using arm-swing navigation intended to feel most like walking. Participants navigate a series of wandering paths and colorful signposts to guide them to 6 clearings, each containing a different activity. See Figure 2 for screenshots during gameplay for each of the 6 activities. In the riverboat activity, players set paper *feeling* boats into the water. Each boat is labeled with a different negative emotion (emotional clarity/cognitive defusion). In the painting activity, players are able to paint on a surface, with their painting slowly disappearing after a short period of time (artful mindfulness). In the scavenger hunt, players search for rocks hidden around the clearing and place them on stumps when found. Each rock is labeled with a positive word, and when all rocks are found, they change color (visual attention/positive affirmations). With bird search, players search for 3 hidden birds by following the bird’s call. When found, the bird flies to a stump, and when all 3 birds are found, flowers appear (auditory mindfulness). In the rabbit hole activity, players stand still near a stump, and rabbits appear the longer they are standing there (attention/awareness). With rock stacking, players stack rocks on each other to create stacks or other formations (attention/focus). See Multimedia Appendix 1 for a video illustration of the environment.

### Ethics Approval

The University of Washington Institutional Review Board approved this study (Study 00003795: Virtual Relaxation Experience).

### Recruitment, Screening, and Enrollment

A convenience sample of teens was recruited from April 2021 through June 2021 via social media (Facebook, Twitter), listservs, and snowball sampling. Eligibility criteria included (1) ages 14 years to 18 years, (2) able to speak and read English, (3) have a smartphone with SMS text messaging capabilities, and (4) currently live in the greater metro area. Prospective participants with a diagnosed seizure disorder were excluded from this study due to the risk of VR triggering a seizure.

Research Electronic Data Capture (REDCap) [37], a secure web-based data collection and management system, was used for all participant survey data collection. Prospective participants accessed the REDCap study eligibility screening survey via a QR code or weblink, which described the study aims and associated activities. Eligibility was automatically determined after the individual completed the survey. Those who were eligible were consented within the REDCap system. Participants were informed of the study procedures and phases and that they could disengage at any time. Parents of participants younger than 18 years were sent an email with study contact information.

Upon entering the study, a VR kit was delivered to the participant’s home via study staff using a no contact protocol described in a previous study by Sonney et al [38]. Participants were emailed an enrollment and orientation packet that included a video demonstration of setting up and charging the VR headset. During the study, research staff used text messaging to check in with participants the day they received the VR kit, on day 3, and once a week thereafter. Participants were informed to contact study staff by text if they had any questions.

### Instrumentation

Surveys included an investigator-developed questionnaire capturing participant age, grade in school, and prior experience using VR (never, once, 2-5 times, ≥6 times) for numerous VR technologies, including standalone headsets, full headsets, and Google cardboard. Participant gender, racial, ethnic, and tribal (if applicable) identities were collected via a write-in option. Standardized surveys focused on implementation outcomes (Aim 1, RQ1) were administered postintervention (Time 2) and included the Acceptability of Intervention Measure, Feasibility of Intervention Measure, Appropriateness of Intervention Measure [39], System Usability Scale (SUS; ɑ=.85) [40], and Intervention Usability Scale (IUS) [41]. Surveys focused on mental health outcomes (Aim 2, RQ3) included the PHQ-9 (ɑ=.89) [42], PSS (ɑ=.71-.91) [43], CFQ (ɑ=.93) [44], and MAAS (ɑ>.80) [45]. These were administered at intake (Time 1), after the 3-week intervention (Time 2), and 4 weeks postintervention (Time 3). Intake surveys were estimated to take 15 minutes to 20 minutes to complete, and exit surveys were estimated to take 15 minutes to 25 minutes.

Momentary instruments (Aim 2, RQ4) included a 5-time per day momentary survey using SEMA 3 software [46] to gather within-day momentary assessments of stress and mood over the 3-week intervention period. Participants were sent 5 scheduled, within-day mood surveys each day of the 3-week study. Within-day surveys asked 2 questions: “How stressed do you feel right now?” and “How sad do you feel right now?” Participants answered these questions on a sliding scale ranging from no sadness/no stress (0) to extremely sad/extremely stressed (100). In addition, a pre- and post-VR survey was developed that included the same stress scale as the within-day survey. However, it also included an abbreviated Positive and Negative Affect Scale [47] and a comfort scale for post-VR: “How comfortable was your VR session?” (1: not very comfortable to 5: very comfortable). Momentary surveys were estimated to take between 30 seconds and 90 seconds to complete.

Given the potential for participants to forget the self-initiated VR survey, we addressed Aim 1, RQ2 using 2 VR use statistics: VR use frequency (number of sessions) and VR use duration (length of use). These were measured through VR activity metrics downloaded directly from each headset. An analytics system built into RESeT saved a VR use log file with a time stamp and duration for each session. In case the analytics system malfunctioned, the Android OS UsageStatsManager was used to retrieve total app use time from the last 24 hours, 7 days, and 30 days.

Finally, to address Aim 1, RQ2, a semistructured interview examined the participant experience, perceived effect, and feedback related to the protocol and environment (Aim 1). For example, “What did you feel using RESeT?” and “What concerns do you have about using RESeT?” Interviews lasted approximately 15 minutes and were recorded via videoconferencing software. In addition, we asked participants about their likelihood to continue using the environment as well as how they might change the design. The semistructured interview took between 10 minutes and 20 minutes to complete.

### Intervention Procedure

Participants were delivered a Quest 2 headset, charger, and customized user manual that included links to troubleshooting and an unboxing video directly to their home. At the start of the study, they were asked to use RESeT for 10 minutes to 15 minutes 3 to 5 times per week. Participants were not further prompted to use the headset nor were they incentivized based upon the amount of headset use during the study. Before each use, they were asked to complete the SEMA presession surveys (stress, affect), explore the RESeT however they desired, and complete the SEMA postsession surveys (stress, affect, comfort). The study team completed weekly check-ins via text message throughout the 3-week pilot and were available for any questions.

At the end of the 3-week pilot, REDCap automatically sent participants the postintervention survey queue, and the study team conducted a semistructured exit interview and scheduled a time to retrieve the VR study kit, which was sanitized and prepared for the next participant. A 3-week follow-up survey queue was automatically sent by REDCap. Participants received digital gift cards after each survey completion. Participants received US $25 at Time 1, US $75 at Time 2, and US $50 at Time 3.

### Data Analysis

#### Quantitative Analyses

All statistical computations were performed in the open-source R software program [48]. Descriptive statistics were used to describe the sample characteristics, survey scores, and intervention use. Longitudinal spaghetti plots were performed to explore time trends and form. A repeated measures ANOVA was conducted to detect group and within-individual differences during survey time points (Time 1-Time 3). Longitudinal mixed effects models with random effects for time, frequency, or duration were computed to detect changes in stress within individuals. A linear regression model with random effects controlled for participant variations was used to measure change in affect and stress resulting from each VR session.

Several models were run to explore whether dosage (number of sessions and duration of use) moderated the change in momentary stress over time. For dosage models, we explored whether changes in stress scores were linear (uniform rate of improvement as dosage increased), quadratic (declining rate of improvement as dosage increased), or cubic (declining and then increasing rate of improvement as dosage increased).

Full maximum likelihood estimation was used, and model building followed a standard procedure [49]. A null model was built first to establish baseline variance for stress over time, followed by longitudinal trend models testing linear, quadratic, and cubic longitudinal trends. These were run on 3 separate sets of models testing longitudinal trends for variables representing number of days since baseline, number of times the VR was used, and number of minutes the VR was used. The best longitudinal trend was selected by model fit deviance statistics using –2 log likelihood and Akaike information criterion. We tested for possible moderation effects by computing models with each variable and simple interaction between them. Moderation models included possible moderation between stress over time and (1) frequency x duration, (2) baseline PSS, (3) baseline PHQ-9, and (4) baseline MAAS. Significance for moderator terms was determined by *t* score significance values for covariate estimates.

#### Post Hoc Groupings

For the purposes of analysis, participants were grouped into depression level and VR use categories based upon their study data. We based 4 mutually exclusive VR use groupings (minimal [<3 sessions], low use [3-4 sessions], moderate use [5-7 sessions], high use [≥8 sessions]) upon their self-administered VR use during the study. Depression levels were assessed using the Time 1 PHQ-9 scores. Participants were grouped into mild (PHQ-9<5), moderate (PHQ-9=5-9), and severe (PHQ-9≥10) depression levels. We excluded 8 participants from the analyses exploring the effect of the VR environment on surveyed variables (depression, retrospective stress, mindfulness, cognitive fusion) given their low use of the VR headset (<3 sessions). However, all 44 participants were included in the analyses exploring the relationship between VR use and stress over time.

#### Qualitative Analyses

As part of our mixed methods design, the qualitative data were gathered concurrently with our quantitative instruments and then explored to help contextualize our quantitative findings. Exit interview data were analyzed using a collaborative thematic analysis protocol. Using raw video data from each of the exit interviews, participant VR session logs, and text messaging, the team of researchers engaged in a collaborative, reflexive thematic analysis [50,51]. Analysis began with the extraction of excerpts that felt salient in relation to our research questions around activity in VR, emotional experience, and the effect of VR on stress. From a review of extracted excerpts, open coding was used to create a categorical code book. With the code book, researchers then revisited the data to further contextualize the categorical codes in an effort to represent the depth and breadth of experiences described by participants. This process was repeated until the research team felt we had sufficient evidence to contextualize our study findings.

## Results

### Participant Characteristics

A total of 118 individuals accessed the eligibility screening, 100 completed the screening, 94 were eligible, and 51 enrolled. Reasons for ineligibility included age older than 18 years (n=4), age younger than 14 years (n=1), seizure disorder (n=1), and out of the geographic area (n=1). After data collection, 7 participants were removed from those enrolled: 2 participants did not complete baseline surveys; 2 did not use the intervention (8% attrition); and although they completed all study procedures and described using the intervention, we found no headset data for 3 participants. Therefore, because we could not objectively confirm use of the headset, these participants were removed from the study. The final sample included 44 adolescents. See Multimedia Appendix 2 for all participant demographic characteristics.

Participants were aged 14 years to 18 years (mean 15.82 years) and in grades 8 through 12 (mean 10.09). Participants identified their gender as boys (n=17), girls (n=23), and nonbinary or gender fluid (n=4). Participants completed a total of 1651 (mean 45.86, SD 19.24) momentary reports of their stress level and mood during the 3-week study period. The average response rate for random, within-day reports was 33%, which was expected given the randomized schedule. In addition, participants completed 330 pre- and post-VR session logs reporting their stress and affect before and after headset use as well as their comfort level.

### RQ1: How Did Teens Use RESeT and How Did They Rate Its Implementation?

Without prompting or using incentives, teens used RESeT, on average, twice a week (number of sessions: mean 6.29, SD 4.51; range 1-24) over the 21-day study period. The average duration of a VR session was 11.5 (SD 6.47; range 1-45) minutes. No significant differences were found when exploring the effect of gender, age, or depression level on VR use nor did we find that VR use affected cognitive fusion, mindfulness, or retrospective stress. We did create post hoc VR use groupings for comparison in our stress analyses. See Table 2 for VR use groupings.

The average usability rating of the VR system (SUS) was good (mean 74.87, SD 11.61); 37 of the 44 participants (84%) rated the system a 68 or higher, suggesting the standalone headset was fairly easy to use without any external support. The average usability rating (IUS) of RESeT was also good (mean 76.92, SD 11.7); 35 of the 44 participants (80%) rated the intervention a 68 or higher, which suggests the VR environment was also fairly easy to use. Intervention acceptability (mean 15.67, SD 3.7), feasibility (mean 15.9, SD 2.95), and appropriateness (mean 15.23, SD 2.99) were all in the high range, suggesting participants found the RESeT content to be appropriate and acceptable. In their session logs, teens reported the environment was moderately comfortable, but reports ranged across the sample (mean 3.43, SD .992).

When asked about the future of RESeT, teens suggested that the VR environment would be most accessible and useful as a school or public library resource, allowing teens to use it on site or to check out the headset for home use when needed.

**Table 2:** Virtual reality use groups by gender and depression level.

Use (number of sessions)	Gender, n (%)				Depression level (via PHQ-9^a^), n (%)		
	Boys (n=17)	Girls (n=23)	Other (n=4)	Mild (<5; n=13)		Moderate (5-9; n=11)	Severe (≥10; n=20)
Minimal (<3; n=8)	2 (5)	5 (11)	1 (2)	1 (2)		2 (5)	5 (11)
Low (3-4; n=8)	4 (9)	3 (7)	1 (2)	3 (7)		5 (11)	0
Medium (5-7; n=15)	6 (14)	7 (16)	2 (5)	5 (11)		1 (2)	9 (20)
High (≥8; n=13)	5 (11)	8 (18)	0	4 (9)		3 (7)	6 (14)

^a^ PHQ-9: Patient Health Questionnaire 9.

### RQ2: What Is the Experience With Using RESeT?

#### General Experience

In their VR Logs, teens reported a small reduction in negative affect, a slight increase in positive affect, and a significant decrease in momentary stress. See Table 3 for full details.

In general, teens enjoyed their experience with the VR environment. Most reported that they liked the audio and visual design. “RESeT helps you chill out with ambient noises and being able to explore” (P1, boy, 16 years old, text message). Overall, teens who engaged in each of the activities most commonly reported liking the rock stacking activity. The least favorite activity was the painting activity.

I really liked the rock stacking. I thought that was nice because you could move the rocks and you could stack them and other places in the area...And, I was a little disappointed by the painting.

P95, nonbinary, 18 years old, exit interview

We analyzed text message and exit interview data to understand how participants described their experience with RESeT. As a result, 4 key themes emerged: calm, regulating, forget about everything, and some discomfort. Each of these themes is described in detail in the following sections.

**Table 3:** Key survey outcomes over the study period.

Survey outcomes	Baseline (n=44), mean (SD)	Postintervention (n=44), mean (SD)	3-week follow-up (n=44), mean (SD)	*F* statistic (*df*)	*P* value
Perceived stress	19.7 (5.7)	18.5 (4.1)	17.4 (7.5)	1.9 (2)	.15
Cognitive fusion	28.5 (7.5)	26.9 (7.3)	24.8 (9.1)	0.4 (2)	.65
Depression	9.0 (4.8)	5.8 (4.4)	6.7 (5.4)	1.0 (2)	.38
Mindfulness/attention	3.9 (0.8)	4.2 (0.5)	4.2 (0.8)	1.2 (2)	.31

#### Calm

Overall, teen participants in the study felt generally positive about the environment. Teens described the environment as “calming,” “relaxing,” and “stress-relieving.” As one teen described:

Kind of makes you calm as a whole—more calm, more relaxed.

P103, boy, 17 years old, exit interview

Another teen described the following:

I really...definitely noticed feeling a lot more calm and mindful after being in the environment and I thought it definitely has a positive impact, especially when you are depressed or anxious about something.

P80, boy, 14 years old, exit interview

#### Regulating

In exit interviews, teens suggested that the VR environment helped them to regulate their moods when overwhelmed or upset. As one teen described:

Mostly I would use it when I was like really upset. And so it helped a lot there. I kind of looked forward to doing it. It changed the way I felt, like mentally and stuff.

P39, 15 years old, nonbinary, exit interview

Some teens also described emotional regulation or making space for them to process big feelings as a result of the environment, for example:

It has 100% the ability to lower and kind of simmer any really high feelings of emotion.

P96, boy, 16 years old, exit interview

#### Forget About Everything

Some teens described it more as a form of escape and distraction from unwanted thoughts or feelings, such as:

It gives teens a space to relax and calm down and just forget about everything happening in the real world.

P116, boy, 15 years old, exit interview

Some participants mentioned feelings of escapism through the VR environment and that it was a great, but temporary, distraction from current stressors:

RESeT let us transport into another world and I forgot about the environment I was in. It transferred me to a different location. It felt like I was going on a trip so even though I had my worries in my head it felt good to forget about the physical things around me and be somewhere else. I'm going on a trip for 10-15 minutes and it relaxes me.

P71, girl, 16 years old, exit interview

#### Some Discomfort

It is important to note that, for some participants, there was some discomfort with the headset causing some nausea or dizziness (n=8) or eye strain (n=3):

Well, when I first started using it, afterwards it hurt my eyes for a little bit, but then, after a while on my eyes got adjusted to it so didn't hurt anymore.

P25, girl, 14 years old, exit interview

Nausea was typically associated with locomotion. Some described the arm swing motion as nauseating, whereas for others, teleporting induced nausea. Overall, nausea and eye strain seemed to lessen or fully resolve by adjusting the headset or getting used to using VR.

Two participants mentioned headaches as a result of using VR, particularly regarding the arm swing movement causing nausea. There was also some comparison of the environment with a video game, which may explain participants feeling bored when compared with entertainment expectations for a video game:

When I first started using the VR headset I kept on getting giant headaches and got really dizzy because I wasn't used to how VR feels yet. So at that point if I weren't doing a study I personally wouldn't continue using the headset.

P38, girl, 18 years old, text message

### RQ3: What Effect Does RESeT Use Have on the Surveyed Measurement of Retrospective Stress (PSS), Depression (PHQ-9), Cognitive Fusion (CFQ), or Mindfulness (MAAS)?

An analysis of surveyed outcomes demonstrated no significant effect of using the VR environment on retrospective stress (PSS), depression (PHQ-9), cognitive fusion (CFQ), or mindfulness (MAAS) for participants regardless of the amount of VR use. See Table 3 for details. However, we did see significant changes in momentary stress and affect, as described in the following sections.

### RQ4: What Effect Does RESeT Use Have on Momentary Stress and Mood?

When exploring the pre- and post-headset session surveys, teens reported a significant reduction in negative affect, a significant increase in positive affect, and a significant reduction in momentary stress. See Table 4 for full details.

Mixed effects models of change in momentary stress over the 21-day study period found that a linear time model was a better fit than quadratic and cubic models. Stress decreased by 0.328 points per day over the study (SE=0.12, *P*=.008).

**Table 4:** Effect of virtual reality (VR) sessions on momentary affect and stress (n=44; 330 reports).

Affect and stress	Before, mean (SD)	After, mean (SD)	Estimates (relative to “before” survey)	*P* value
Negative affect	9.2 (3.6)	6.9 (2.4)	–2.3	.001
Positive affect	10.8 (4.0)	12.0 (4.1)	1.3	.001
Momentary stress	37.9 (27.1)	24.0 (20.6)	–13.3	.001

### RQ4a: Do Baseline Depression, Mindfulness, and Stress Moderate Momentary Stress Over the 3-Week Intervention?

The rate of decrease in momentary stress score was not significantly moderated by baseline scores of retrospective stress (PSS: interaction=–0.021, SE=0.021, *P*=.31), depression (PHQ-9: interaction=–0.04, SE=0.03, *P*=.14), or mindfulness (MAAS: interaction=0.14, SE=0.16, *P*=.38).

### RQ4b: Does Dosage (Frequency and Duration of VR Use) Moderate Momentary Stress Over the 3-Week Intervention?

**Figure 3:**
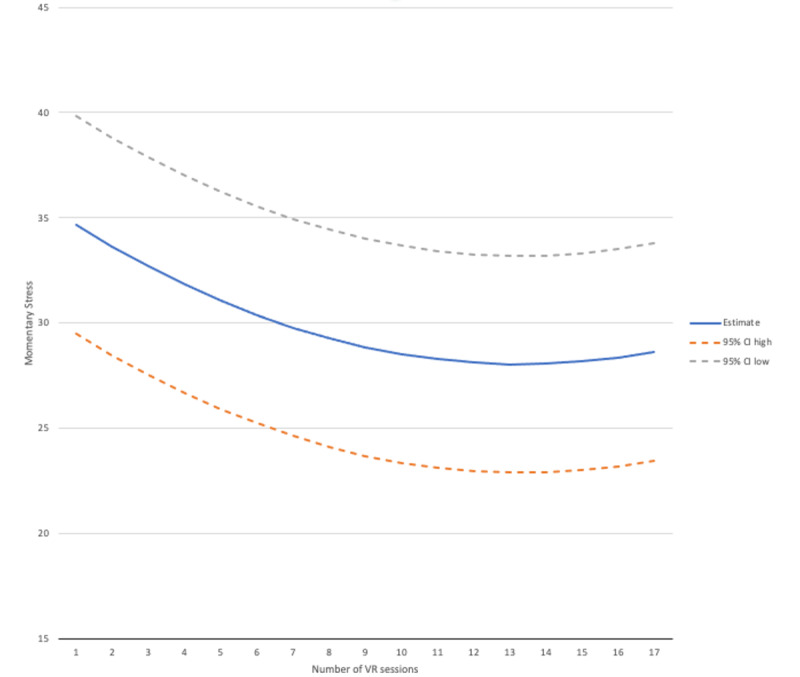
Predicted change in the stress score, with the 95% CI, by number of uses of the virtual reality (VR) headset over the 3-week period.

The best-fitting mixed effects model for frequency of use included both linear and quadratic use frequency, indicating that there was a stronger association with improved stress scores during the first several uses (linear frequency=–1.08, SE=0.50, *P*=.03), which decreased as frequency of use increased (quadratic frequency=0.043, SE=0.02, *P*=.03; see Figure 3). The best-fitting model for cumulative duration of use was linear, though the individual parameter for duration was not significant (linear minutes=0.01, SE=0.003, *P*=.07). A model exploring a possible interaction effect of frequency by duration on stress did not find significant effects (frequency x duration=0.001, SE=0.001, *P*=.61). See Figure 3 for an illustration.

## Discussion

### Principal Findings

It was evident from this study that the VR environment was desirable and a usable system within a self-administered, autonomous home setting. In addition, teens chose to use RESeT repeatedly of their own volition despite its limited interactions. As with our previous studies [28,32], teens confirmed their desire and enjoyment of nature in VR as a stress-reducing environment.

Although RESeT did not significantly affect repeated survey measurements of depression, mindfulness, nor cognitive fusion, it did decrease momentary stress and positively affect momentary mood. This reduction in stress was correlated with the frequency of use, suggesting that self-administered VR environments may be effective to reduce momentary stress. However, the benefit from increased frequency of use tapered off with high use, suggesting that there may be a limit to the impact of RESeT over time. Counterintuitively, although frequency of use was associated with further stress reduction, duration of use was not, suggesting that longer sessions may not be more beneficial. Also likely, it could be that participants self-regulated their use and ended a session once they felt a reduction in stress, such that long sessions and short sessions had a relatively equal impact.

### Comparison With Prior Work

It was not surprising that more stable factors such as depression, cognitive fusion, and mindfulness were not significantly changed by this short-term, self-administered intervention. However, our exit interviews with teens provided strong evidence that, for some teens, the RESeT VR environment provided a place for relaxation as well as emotional regulation. Bond et al [52] found that VR allowed for “safe space to practice” interactions for adults with agoraphobia. It is feasible that VR can also provide a safe space to process big emotions for teens. Although we hoped to see significant changes in cognitive fusion given one exercise (the boat launch) was designed specifically for this purpose, teens did describe experiences of emotional regulation. Computer games have shown significant improvements in emotional regulation in adults [53], and a preliminary study found VR could both improve and measure emotional regulation in adolescents [54]. For this reason, measuring emotional regulation as a result of VR nature environment experiences may be an important factor to consider.

Previously, we conducted a 2-week study exploring a high-fidelity, commercial VR nature-based environment [19]. In the 2-week study, momentary stress was also significantly reduced, suggesting that these types of environments may be effective to reduce momentary stress. In the 2-week study, we also found participants averaged about 6 VR sessions over the study period. This suggests that, perhaps in these self-administered environments, engagement or effect may taper around 6 sessions. Interestingly, similar themes of relaxation and escape emerged from the participants’ responses to the VR environment. Granted, both studies explored nature settings, but combined findings suggest teens find nature relaxing and that VR offers an opportunity to escape real-world stressors. Future design research is needed to explore further translation of evidence-based CBT, dialectical behavioral therapy, and acceptance and commitment therapy exercises into immersive VR activities in an effort to understand what types of activities are most engaging and effective.

### Limitations and Next Steps

This study was an exploratory usability study of self-administered VR and therefore does not constitute a more rigorous clinical trial. In addition, this study was limited in several ways. First, to establish the effectiveness of RESeT, a control or comparison group, possibly using a well-crafted placebo VR experience, would be necessary. Second, this study was limited in the diversity of its sample (broad range of depression levels) as well as participants’ self-selection and ability to self-administer the VR tool. Although each of these factors provides some real-world context about how VR may be used by teens of varying levels of depression, it also limits any generalizability of these data to other samples. Third, the study also lacks controlled data or comparison data to determine the true effect of VR on the measured variables. Fourth, the participant-level sample size was modest and lacked statistical power to detect small to moderate effects; findings should be viewed as preliminary, consistent with the scope of an exploratory study. Finally, given the probable novelty of VR and short intervention period, teen participants likely experienced a novelty effect, and engagement may have tapered during a longer intervention period.

Further research could explore the VR headset as a community-based tool, such as in a school or library setting, to increase access for teens. VR technology has been found an engaging tool in public library settings [55], which has prompted some public libraries to launch programs to support mental health [56]. As per Kelly et al [57], we included minimal interactions, hoping not to distract participants from mindfulness. However, teens wanted more engagement and interaction; therefore, future evidence-based mental health VR interventions could explore more engaging and interactive activities, as it is likely with a larger platform, more choices, and a more diverse set of activities, teens would be more engaged with the VR platform.

### Conclusion

Teens enjoyed and repeatedly used our VR environment, which included 6 evidence-based activities in an open world nature environment, without further incentive. They found the VR system and the RESeT usable, and many reported feeling relaxed or calm as a result. We found that stress decreased over time and that increased VR session frequency further decreased momentary stress. However, we also learned that increased session duration did not improve stress outcomes, suggesting that even brief VR sessions can be effective. These findings indicate that VR is a feasible and likely attractive platform for evidence-based mental health interventions.

## Appendix

Multimedia Appendix 1 RESeT VR Environment Demonstration.

Multimedia Appendix 2 Participant Demographics.

## Abbreviations

CBT: cognitive behavioral theory
CFQ: Cognitive Fusion Questionnaire
IUS: Intervention Usability Questionnaire
MAAS: Mindfulness Attention and Awareness Scale
PD: participatory design
PHQ-9: Patient Health Questionnaire 9
PSS: Perceived Stress Scale
REDCap: Research Electronic Data Capture
RESeT: Relaxation Environment for Stress in Teens
RQ: research question
SG: serious game
SUS: System Usability Scale
VR: virtual reality

## Data Availability

Study data are available upon request.
