# Supplementary material for: Using Virtual Reality to Reduce Stress in Adolescents: Mixed Methods Usability Study
Source: JMIR XR Spat Comput. 2024 Apr 22;1:e49171. doi: 10.2196/49171 (PMC12671315; doi:10.2196/49171)
Supplement: Multimedia Appendix 2 [file xr_v1i1e49171_app2.docx]

**Multimedia Appendix 2. Participant Demographics**

| **Gender** | **n** | **%** | **Ethnicity** | **n** | **%** |
| --- | --- | --- | --- | --- | --- |
| Girls | 23 | 52% | White/  Caucasian | 13 | 30% |
| Boys | 17 | 39% | Asian/  Asian-American | 5 | 11% |
| Non-Binary/  Gender fluid | 4 | 9% | Vietnamese | 5 | 11% |
| **Ages** | **n** | **%** | Chinese | 4 | 9% |
| 14 | 7 | 16% | No response | 3 | 7% |
| 15 | 13 | 30% | Latino | 2 | 5% |
| 16 | 9 | 20% | Native American | 2 | 5% |
| 17 | 11 | 25% | Pakistani | 2 | 5% |
| 18 | 4 | 9% | Somali | 2 | 5% |
| **Grade** | **n** | **%** | Middle Eastern | 1 | 2% |
| 8 | 3 | 7% | Irish German | 1 | 2% |
| 9 | 15 | 34% | Russian | 1 | 2% |
| 10 | 7 | 16% | African American | 1 | 2% |
| 11 | 13 | 30% | Middle Eastern | 1 | 2% |
| 12 | 6 | 14% | Tibetan | 1 | 2% |
